# Development of the European Healthcare and Social Cost Database (EU HCSCD) for use in economic evaluation of healthcare programs

**DOI:** 10.1186/s12913-022-07791-z

**Published:** 2022-03-27

**Authors:** Jaime Espín, Zuzana Špacírová, Joan Rovira, David Epstein, Antonio Olry de Labry Lima, Leticia García-Mochón

**Affiliations:** 1 Andalusian School of Public Health/Escuela Andaluza de Salud Pública (EASP), Cuesta del Observatorio 4, 18011 Granada, Spain; 2 CIBER en Epidemiología y Salud Pública (CIBERESP), Spain/CIBER of Epidemiology and Public Health (CIBERESP), Madrid, Spain; 3 Instituto de Investigación Biosanitaria ibs, Granada, Spain; 4 University of Granada, Granada, Spain

**Keywords:** Costs, Economic evaluation, Transferability, Database, Costing methodology

## Abstract

**Introduction:**

Costs are one of the critical factors for the transferability of the results in health technology assessment and economic evaluation. The objective is to develop a cost database at the European level to facilitate cross-border cost comparisons in different settings and explains the factors that lead to differences in healthcare costs in different countries, taking into account the differences between health systems and other factors.

**Methodology:**

The core of the database is compounded of three main categories (primary resources, composite goods and services, and complex processes and interventions) organized into 13 subcategories. A number of elements providing as detailed information of unit cost as possible were identified in order to mitigate the problem of comparability. Consortium partners validated both the database structure and selected costing items.

**Results:**

Twenty-seven costing items included in the EU HCSCD resulted in 1450 unit costs when taking into account all item subtypes and countries. Cross-country differences in costs are driven by the type of resources included in the costing items (e.g., overhead costs in case of complex processes and interventions) or by the variety of existing brands and/or models and the type of unit value in most of the primary resources.

**Conclusion:**

The EU HCSCD is the only public unit healthcare and social cost database at European level that gather data on unit costs and explains differences in costs across countries. Its maintenance and regular data updating will enable establishing specific systems for generating and recording information that will meet many of its current limitations.

**Supplementary Information:**

The online version contains supplementary material available at 10.1186/s12913-022-07791-z.

## Background

Economic evaluations (EE) are recognised as a key element of priority setting decisions in Health Technology Assessment. EE should be an unbiased and transparent exercise, and should address issues of generalizability (the degree to which the results of an observation hold true in other settings) [1] and transferability (designing and reporting evaluations in such a way that at least some components can be reusable in other settings) [2]. Transferability can be greatly enhanced by following good practice by estimating and reporting units of resources separately from the unit monetary values −price or unit cost− of those resources.

Hence accurate unit costs are essential inputs for economic evaluations. Unit cost data are available from multitudinous sources, for example: previous literature, national databases, healthcare provider administrative or billing systems, or expert opinion. This diversity of sources can make interpretation and generalizability of economic evaluations challenging. The unit costs of similar-seeming resource items may vary, for example, due to differences in the definition of the unit of activity, the quantity, quality or mix of resource inputs used in providing a service or activity, average price levels across countries and across time, fluctuations in currency exchange rates, or cost-accounting methodology [3]. HTA evaluations need to be adjusted to circumstances in each national (or even regional) context, and sponsors of health technologies or other researchers often attempt to construct transferable evaluations that can be adjusted across countries and jurisdictions, rather than starting a new analysis from scratch [4].

To address these issues, the European Healthcare and Social Cost Database (EU HCSCD) [5] for use in Health Technology Assessment (HTA) across countries has been developed. The remit of EU HCSCD is to provide a freely-available dataset of unit costs of commonly used healthcare resource items across European countries, and builds on previous European-wide projects HealthBASKET [6] and EuroDRG [7]. Existing cross-country unit cost databases require a fee [8], or are designed for strategic health planning in low and middle-income countries (the OneHealth Tool [9]).

Hence the EU HCSCD meets an unmet need among researchers, and it is expected to provide several benefits. First, the EU HCSCD will improve transferability by making it easier to carry out multi-country studies and to adapt economic evaluation studies from country to country, hence saving human resources time and expense in the task of finding appropriate healthcare and social unit costs. Second, the database has information not only on the unit cost, but also describes how that item was constructed or estimated in a common format. This information may help researchers explore and explain variation in unit costs between and within countries. Will improve transparency by presenting unit costs estimated in different countries in a common format, allowing any variation in unit costs to be explored and explained. Third, the availability of this detailed cross-country resource may help national data producers (such as those responsible for Diagnostic Resource Groups) identify strengths and weaknesses in their costing methodology and, over time, may promote the standardization and improvement of these sources .

The EU HCSCD includes both healthcare and social (non-healthcare and productivity) costs. This paper describes the construction and content of the healthcare unit costs module, which was developed in the work package ‘Developing a costing methodology and a core dataset of costs for facilitating cross border comparisons in economic evaluations’ of the IMPACT-HTA project (No 779312) [10].

### Construction and content

#### Structure of the EU HCSCD

The unit costs in the database are classified into three main categories: primary resources (those that cannot be further divided into smaller units, such as cost per hour of professionals or prices of medicines), composite goods and services (bundles of several primary resources that are consumed jointly, such as a day in a hospital ward, or an outpatient visit), and complex processes and interventions (units of activity that aggregate diverse types of resources, such as medical interventions and surgical procedures). Table 1 provides an overview of the categories, subcategories of the 27 items currently listed in the EU HCSCD. Each item may have several fields corresponding to the sources available in each country.

**Table 1:** Structure of the European Healthcare and Social Cost Database: Categories, subcategories and items

Category	Subcategory	Definition	Costing items
Primary (homogenous) resources	Medicines	Drug or other preparation for the treatment or prevention of disease	• Atorvastatin • Paracetamol • Trastuzumab
	Medical devices	Article, instrument, apparatus or machine that is used in the prevention, diagnosis or treatment of illness or disease, or for detecting, measuring, restoring, correcting or modifying the structure or function of the body for some health purpose [11].	• Drug eluting stent • Wearable cardioverter-defibrillator
	Health products/ Disposables	Items designed for single use or those that may be used more than once after proper cleaning and sterilisation and/or disinfection [12].	• Glucose test strips
	Personnel	Labour time of health care professionals (e.g., workers employed in health care institutions or processes).	• General practitioner • Nurse • Specialist
Composite goods and services	Outpatient visit	Visit of a patient who is not hospitalized overnight but who visits a hospital or clinic for diagnosis or non-surgical treatment [13]. Home visits (medical or nursing staff attending a patient at his home) and Accident and Emergency visits were also considered.	• General practitioner visit • Specialist visit • Accident & Emergency visit
	Hospitalization	Admittance to the hospital as an inpatient [13].	• Day of hospitalization at “normal” ward • Day of hospitalization at Intensive Care Unit
	Image diagnosis	Radiography, sonography, and other technologies used to create a graphic depiction of the body for diagnosis or therapeutic purposes [13].	• Ultrasound scan • Computerized Tomography Scan
	Laboratory tests	Services provided by medical laboratories for the diagnosis of disease [13].	• Creatinine • Ferritin
	Ambulance service	Service provided by a vehicle which can transport medical patients to the treatment site or back to their place of residence, and in some instances will also provide out-of-hospital medical care to the patient during the transportation.	• Non-emergency patient transport • Intensive care ambulance
	Diagnostic procedures	Type of test used to help diagnose a disease or condition [14].	• Colonoscopy
	Therapeutic procedures	Medicine or therapy used to cure disease or pain by the involvement and intercession of proactive, therapeutic practice [15].	• Haemodialysis • Oxygen therapy
Complex processes and interventions	Inpatient medical and surgical processes	Require patients to stay the night following the surgery.	• Heart failure (ICD10: 50) • Hernia inguinal, femoral, umbilical (ICD10: K40, K41; K42)
	Day case procedures/ Outpatient surgery	Day case refers to a patient or case that comes into hospital for a surgical procedure and is dealt with and released in the course of one day [16]. Outpatient surgery refers to usually minor invasive interventions performed on ambulatory patients usually in a clinic or consulting room.	• Laparoscopic cholecystectomy • Cataract extirpation

#### Fields describing the EU HCSCD costing items

Ensuring comparability consists of collecting as detailed information on costing methodologies used to estimate cost of each item included in the EU HCSCD as possible. This means the knowledge about what resources are included in the cost, how were the resources estimated and how was the unit cost calculated and assigned to the resources. In the long term, the comparability can only be attained if all countries/institutions use the same accounting methodology. However, the unit values applied at present have different origins, not only cost accounting from health care organization, but also market prices, tariffs, public prices, etc. Therefore, all the fields described below have been included in the cost database to mitigate the problems of comparability (Table 2).

**Table 2:** Examples of costing items included in the European Healthcare and Social Cost Database (EU HCSCD)

Field	Description	Example 1	Example 2	Example 3
Category		Primary resources	Composite goods and services	Complex processes and interventions
Subcategory		Medical devices	Image diagnosis	Inpatient medical and surgical processes
Costing item		Wearable cardioverter-defibrillator	Computerized tomography scan	Heart failure (ICD10: I50)
Item subtype	Provides further description of the costing item.	n.a.	Scan of the skull and its contents, without contrast medium	Very complex
Item in local language	Apart from English, each item is provided in the language of the country the resource is taken from.	*Defibrillatore Indossabile*	*Scanographie du crâne et de son contenu, sans ijection de preduit de contraste*	*Hjärtsvikt & chock*
Code	Combination of letters and/or numbers each costing item is described with in the original source.	Z12030503	ACQK001	E47A
Model	Value used to represent one medical device or a family of medical devices to group many variations that have shared characteristics. This field applies to medical devices and health products/disposables.	WCD-4000	n.a.	n.a.
Brand	Proprietary/commercial name occasionally used to assist in the identification of the regulated medical device and/ or disposables/ reusable. This field applies to medicines, medical devices and health products/ disposables.	Zoll Medical Italia SRL	n.a.	n.a.
Country	The country the data originated from.	Italy	France	Sweden
Region		Sardinia	n.a.	Östergötland
Type of unit	The way the units are delivered (e.g., box). This field applied to medicines and health products/ disposables.	n.a.	n.a.	n.a.
Number of units	The number of items delivered in the year of observation that are included in unit price. The purpose of this field is to know how reliable a certain value –usually an average − is.	1	1	1
Unit of measurement	The unit that can be acquired at an observable price or the unit that is used by each health care centre/hospital.	Wearable cardioverter-defibrillator (WCD)	Procedure	Process
Strength	The amount of a drug in a given dosage form, measured as the number of micrograms per millilitre. This field applies only to medicines.	n.a.	n.a.	n.a.
Year	Refers to the year of cost publication.	2019	2018	2019
Local price	The unit price of each item.	3700	25.27	72,198
Local currency	The currency of the unit price of the country the items refers to.	Euro	Euro	Swedish krona
Price in euros	Local price converted into euros.	3700	25.27	6909.35
Year (GDP deflator)	Year of the last GDP deflator available. The GDP deflator is country-specific, but the last value available comes from the same year.	n.a.	2019	N.a.
Local price (GDP deflator applied)	Local price after applying the last country-specific GDP deflator available.	3700	25.58	72,198
Price in euros (GDP deflator applied)	Local price (GDP deflator applied) converted into euros.	3700	25.58	6909.35
Year (CPI)	Year of the last CPI available. The CPI is country-specific, but the last value available comes from the same year.	n.a.	2019	n.a.
Local price (CPI applied)	Local price after applying the last country-specific CPI available.	3700	25.60	72,198
Price in euro (CPI applied)	Local price (CPI applied) converted into euros.	3700	25.60	6909.35
Type of unit value	Type of monetary value that is placed on each of the resources used.	Leasing price	tariff	Tariff
Type of institution	Type of centre where the patients were attended.	Hospital	Ambulatory care	Hospital
Source of unit cost data	The institution that published the data, that is, the institution responsible for communicating or publishing the costing information.	*Sistema Sanitario Regione Sardegna*	*Securité Sociale l’Assurance Maladie*	*Sydöstra sjukvårds-regionen*
Reference of unit cost data	A web page where the publication, report or database with the unit cost is mentioned.	https://www.aobrotzu.it/documenti/9_204_20190124111	https://www.ameli.fr/accueil-de-la-ccam/index.php	https://plus.rjl.se/infopage.jsf?nodeld=41089
Notes	Contains any explanations or notes relevant to the costing methodology, if available (i.e. what cost elements are included in the unit cost, how are these elements identified –micro-costing or gross-costing−, how are cost elements valued –top-down or bottom-up−, etc.	Cost per 1-month lease. The code refers to CND (*Classificazione Nazionale dei Dispositivi medici*).	Consultation cost is not included. Payer’s perspective. There are the following extra charges: 40€ if the procedure is performed urgently on Sundays and public holidays, 80€ for paediatric night urgency 12–8 am, 50€ for emergency except paediatricians, + 49% for patients < 5 years old, + 15.8% for major act radiography carried out by a radiologist, a pulmonologist or a rheumatologist.	The tariff is based on DRG. Costing methodology used in identification and valuation of resources: top-down micro-costing. Depreciation of building and financial cost are included. Research cost and teaching cost are excluded.

*GDP* Gross Domestic Product, *CPI* Consumer Price Index

#### Data collection

Searches were carried out on the health organizations’ websites (e.g., EUnetHTA, NHS England, European Observatory on Health Systems and Policies, Department of Health (London), Centre of Health Economics (University of York), etc.) and the most prevalent diseases worldwide were checked in order to decide which costing items (interventions, medicines, medical devices, etc.) are among the most used in economic evaluations and, therefore, should be included in the beta version of the database. At least one costing item was selected within each costing subcategory. Thus, 27 costing items were included in the EU HCSCD, which resulted in 1450 costs when 9 countries (England, France, Germany, Italy, Poland, Portugal, Slovenia, Spain and Sweden) and all item subtypes were taken into account [5]. The next step consisted of identifying official cost databases containing these items and any accompanying publicly available documents describing the cost-accounting methodology used to construct them. These were obtained from websites of health authorities and health ministries in each country. These sources of material are listed in Supplementary Material.

#### Validation of data

The consortium partners (London School of Economics, Bocconi University of Milan, Institute for Economic Research of Slovenia, Assistance Hôpitaux Publique de Paris (AP-HP), Instituto Superior Técnico (IST) of Portugal, Dental and Pharmaceutical Benefits Agency (TLV) of Sweden, Agency for Health Technology Assessement and Tariff System (AOTMiT) of Poland and University of Bielefeld of Germany) verified this material and provided references to any additional relevant databases and documentation. All documentation was translated to English with the assistance of online tools and consortium partners. An online workshop was organized at the end of October 2019 where one consortium partner from each country explained the functioning of their respective national database and provided any comments on the costing methodology. Consortium partners checked and validated the final selection of data items before inclusion in the EU HCSCD.

#### Annual rate of change in average price levels in the country

We updated values to 2019 prices using Gross Domestic Product (GDP) deflator [17] and Consumer Price Index (CPI) [18] for each country. The WHO-CHOICE guide to cost-effectiveness analysis recommended using GDP price deflators (with the CPI measure being the next best alternative) [19, 20]. GDP deflator or CPI are used to update prices and costs to the current year in the local currency.

#### Conversion of local price into euros

The conversion is based on rates taken from the European Central Bank webpage [21] and is made as follows. The unit price of a particular medical procedure corresponding to let us say England year 2014 is introduced in the database in local currency, in this case in British pound. The conversion to euros is made according to the last conversion rate introduced in the database for the same year, which is the last day of December. In case in some country a cost of a current year needs to be introduced in the EU HCSCD, the conversion rate of that country corresponds to 1st January of the same year. At the end of the year, this conversion rate is updated to the last day of the same year and all costs corresponding to that year are automatically updated. Note that local price and price in euro refer to the same year.

#### Access and use of the database

The free-access database is available at https://www.easp.es/Impact-Hta/default [5]. There are two options: healthcare cost and social cost. Click on healthcare cost and then click on ‘Go to the Cost Database’. Three searching strategies have been defined: basic search, view all costs and advanced search. Basic search consists of searching the typed word across the field ‘Item’. E.g., if a user types ‘general practitioner’, the database will report all the items containing ‘general practitioner’. Together with the searched item, seven additional fields are displayed: Item subtype, Unit of measurement, Year, Country, Price in euro, Price in euro (GDP Deflator applied) and Price in euro (CPI applied). All the information regarding a particular costing item is displayed after clicking on the button ‘view’. This has to be done for item separately. To visualize all the information for all the items found, the users can select all these items and then click on ‘export csv’. The search option ‘view all costs’ allows users to visualize all costs introduced into the database. The modality ‘advanced search’ is suitable for those users who want to search across the particular fields of interest. By clicking on ‘advanced search’, all the fields related to a particular cost item are displayed.

#### Maintenance of the database

Amplifying the number of costing items and updating the costs in the future years are issues that need to be discussed. Even though all the costs can be updated with GDP deflator or CPI in future years; this would be futile and would quickly make the database irrelevant. The only way the database will remain relevant is if the costs are substitutes by their actual values from the actual source data each time they are updated in the original sources. Even the funding from the European Union’s Horizon 2020 research and innovation program (grant agreement No 779312) expired by the end of June 2021, there is still a need of having such a database. The team members are currently searching for more funding in order to maintain the database. If funding becomes available, all users would propose amendments (e.g., updates to items or additional items). Those users that apply for it (ideally a couple of them from different countries) will have the power to submit the data to the database. The principal administrator (a person from the IMPACT-HTA project team) will be in charge of revising them and making the updates periodically. All changes will be notified to the users by email. Those users wishing to be in the mail list can send an email to the Principal Investigator Professor Jaime Espín (jaime@easp.es). Each time a new item is proposed, subscribed users will receive a notification on searching for that item in their country and send it to the above contact. Once this new item is introduced into the database, the users will receive a notification. In the absence of financing, the authors propose that a community of volunteers (users) through a model of open collaboration maintains the database.

This is a Beta version, which is being released now for testing and feedback from the health economic community to inform further development and maintenance, therefore any comments and suggestions from users are welcomed. Anyone is free to use the data without restriction, but are asked to cite this article.

## Discussion

### Strengths and limitations

The EU HSCSD has been constructed with the aim of improving transparency, generalizability and transferability of economic evaluations. This resource should help researchers to find unit cost data quickly, cheaply and accurately. It has been developed with a user-friendly design and is relatively simple to manage and update. Where current year data are unavailable, it updates costs using CPI and GDP deflators [19]. Links are provided to the original sources of information. Therefore, even if a researcher needs data for some item that has not been introduced into the database so far, he or she can find it in the original source. The EU HCSCD also provide an useful input for standardized unit cost templates for a multi-sectoral service and resource use measurement instrument developed as part of PECUNIA project to ensure cross-country comparability and transferability [22].

Unit cost data have several limitations that need to be borne in mind when comparing values across countries or considering which values are appropriate as inputs to economic evaluations. As described elsewhere [23, 24], not all countries provide official unit costs for all items, and few countries provide transparent documentation on which types of resources are included in the total cost or how those values were estimated.

Many ‘primary resources’ are likely to be defined in a fairly standard way across countries, for example: medicines by name and composition, devices by manufacturer and model. Healthcare professionals may be more complicated. Health professionals of the same grade may have quite different functions, for example, in some countries; a senior doctor has a dedicated percentage of time for administration, travel or research, but not in other countries. In some countries, nurses can take senior roles (for example, the “consultant nurse” in the United Kingdom), in other countries their role is more restricted. Other variation arises in the unit of measurement of the source (for example: cost per hour, mean cost per patient per year) or type of expenses included (for example: travel or training). Salaries in the health service may not be “market prices” (that is, reflecting relative scarcity) but are often determined by other factors, such as labour union negotiations, national legislation, workers’ seniority, and so on [25–28].

Regarding accessibility of cost data, current databases of official prices of medicines were found in each country, though with some heterogeneity in definition. The official “public” price is published in England, France, Poland, Portugal and Sweden. In Germany, Spain and Slovenia, the reference price is published, while in Italy, it is the ex-factory price [29]. Costs of medical devices, health products and disposables were more difficult to obtain and the data available were rarely up-to-date. The comparison of costs of medicines, medical devices and health products is hampered by the variety of brands and models. The prices quoted for medical devices and health products included leasing price, ex-factory price, public price, purchase price, reference price and wholesale price.

‘Complex processes and interventions’ refers to inpatient and ambulatory surgical procedures. In some countries, costs of ambulatory procedures can be found in the same database as hospital admissions. For example, in England some ambulatory procedures are classified as Diagnosis-Related Groups (DRGs) (where they are called “day-case admissions”) or, as in Sweden, there is a close relationship between DRG costs and costs of ambulatory procedures. In some countries, tariffs are published for ambulatory procedures, but not costs (Portugal, Italy, Spain). In Italy, tariffs are published at national and provincial levels, while in Spain, there are only provincial tariffs. In France, Germany, Poland and Slovenia, some ambulatory procedures have DRG costs, while others are only attributed a tariff.

The methodology of costing DRGs is often reasonably well documented, though there are some gaps and uncertainties, especially around the treatment of overheads [23]. It is usually less clear how the costs or tariffs of other types of composite services and complex interventions were calculated and what resources were included. For example, a laboratory test is a service that includes some consumables, along with laboratory technician time, administration, communication of results to the patient, etc. The tariff for a given test (for example, a creatine test) might vary depending on whether it is provided in a hospital or in a primary care clinic. Another example is an outpatient consultation. The cost or tariff may vary, depending on where it takes place, whether tests and treatments are included or not, and the type of professional. The cost of an ambulance service attendance may differ across countries because of the unit of measurement (per journey, per intervention, per kilometre, per patient/year, per mobile unit/year, per hour), or the type of service (urban, interurban, transport for dialysis, and so on). The cost of radiographic imaging may depend on the duration, whether contrast was used, the number of areas, whether the patient accessed directly to the service or was referred by his or her primary care doctor and so on.

## Conclusions

The EU HCSCD is the only public unit healthcare and social cost database at European level. The project team are actively working towards setting up a stable consortium responsible for improving, updating and ensuring the continuity of the EU HCSCD. National governments or statistical agencies should review their costing methods in healthcare and ensure that they meet best practice [30, 31]. We also recommend the creation of a European Union Task Force to periodically revise national healthcare classification and costing methodologies at EU level and work towards harmonization and standardization where possible.

## Supplementary Information

**Additional file 1.**

## Data Availability

All data are available at https://www.easp.es/Impact-Hta/.

## Abbreviations

EU HCSCD: European Healthcare and Social Cost Database
EE: Economic Evaluation
HTA: Health Technology Assessment
DRGs: Diagnosis-Related Groups
GDP: Gross Domestic Product
CPI: Consumer Price Index
