# Supplementary material for: Development of the European Healthcare and Social Cost Database (EU HCSCD) for use in economic evaluation of healthcare programs
Source: BMC Health Serv Res. 2022 Mar 27;22:405. doi: 10.1186/s12913-022-07791-z (PMC8962458; doi:10.1186/s12913-022-07791-z)
Supplement: Supplementary file 1 — Additional file 1. [file 12913_2022_7791_MOESM1_ESM.docx]

| **England** | | | |
| --- | --- | --- | --- |
| **Category** | **Subcategory** | **Institution that publishes the cost** | **Link to the website where the cost is published** |
| Primary resources | Medicines | British National Formulary (BNF) | <https://www.nice.org.uk/bnf-uk-only> |
|  | Medical devices | National Institute for Clinical Excellence (NICE) | <https://www.nice.org.uk/guidance/ta152/resources/drugeluting-stents-for-the-treatment-of-coronary-artery-disease-pdf-82598311384261> |
|  | Health products/ Disposables | NHS Business Service Authority | <https://www.nhsbsa.nhs.uk/sites/default/files/2020-03/Drug%20Tariff%20April%202020.pdf> |
|  | Personnel | Personnel Social Services Research Unit (PSSRU) | <https://www.pssru.ac.uk/project-pages/unit-costs/unit-costs-2018/> |
| Composite goods and services | Outpatient visit | National Health Service (NHS) | <https://www.england.nhs.uk/national-cost-collection/>  <https://www.england.nhs.uk/pay-syst/national-tariff/national-tariff-payment-system/> |
|  |  | Personnel Social Services Research Unit (PSSRU) | <https://www.pssru.ac.uk/project-pages/unit-costs/unit-costs-2018/> |
|  | Hospitalization | National Health Service (NHS) | <https://www.england.nhs.uk/national-cost-collection/>  <https://www.england.nhs.uk/pay-syst/national-tariff/national-tariff-payment-system/> |
|  | Image diagnosis |  |  |
|  | Diagnostic procedures |  |  |
|  | Therapeutic procedures |  |  |
|  | Laboratory tests | National Health Service (NHS) | <https://www.england.nhs.uk/national-cost-collection/> |
|  | Ambulance services | Personnel Social Services Research Unit (PSSRU) | <https://www.pssru.ac.uk/project-pages/unit-costs/unit-costs-2018/> |
| Complex processes and interventions | Inpatient medical and surgical processes | National Health Service (NHS) | <https://www.england.nhs.uk/national-cost-collection/>  <https://www.england.nhs.uk/pay-syst/national-tariff/national-tariff-payment-system/> |
|  | Day case procedures/ Outpatient surgery |  |  |
|  |  |  |  |

Supplementary Table S1. Sources of unit costs

| **France** | | | |
| --- | --- | --- | --- |
| **Category** | **Subcategory** | **Institution that publishes the cost** | **Link to the website where the cost is published** |
| Primary resources | Medicines | National Agency for the Safety of Medicines and Health products | <http://base-donnees-publique.medicaments.gouv.fr/> |
|  | Medical devices | Social Security Health Insurance | <https://www.ameli.fr/medecin/exercice-liberal/remuneration/consultations-actes/nomenclatures-codage/liste-produits-prestations-lpp> |
|  | Health products/ Disposables |  |  |
| Composite goods and services | Outpatient visit | Court of Audit | <https://www.ccomptes.fr/system/files/2019-02/08-urgences-hospitalieres-Tome-2.pdf> |
|  |  | Social Security Health Insurance | <https://www.ameli.fr/medecin/exercice-liberal/remuneration/consultations-actes/tarifs/tarifs-generalistes/tarifs-metropole> |
|  | Image diagnosis | Social Security Health Insurance | <https://www.ameli.fr/accueil-de-la-ccam/index.php> |
|  | Laboratory tests | Social Security Health Insurance | <http://www.codage.ext.cnamts.fr/> |
|  | Ambulance services | Court of Audit | <https://www.ccomptes.fr/system/files/2019-10/RALFSS-2019-06-transports-programmes-secteurs-sanitaire-medicosocial.pdf> |
|  | Therapeutic procedures | Scansanté | <https://www.scansante.fr/applications/enc-mco> |
|  |  | Agency for Information on Hospital Care | <https://www.atih.sante.fr/tarifs-mco-et-had> |
| Complex processes and interventions | Inpatient medical and surgical processes | Scansanté  Agency for Information on Hospital Care | <https://www.scansante.fr/applications/enc-mco>  <https://www.atih.sante.fr/tarifs-mco-et-had> |
|  | Day case procedures/ Outpatient surgery |  |  |

| **Germany** | | | |
| --- | --- | --- | --- |
| **Category** | **Subcategory** | **Institution that publishes the cost** | **Link to the website where the cost is published** |
| Primary resources | Medicines | German Institute for Medical Documentation and Information | <https://www.dimdi.de/dynamic/.downloads/arzneimittel/festbetraege/2020/festbetraege-20200901.pdf> |
|  |  | Institute for Hospital Remuneration System | <https://www.g-drg.de/content/download/7388/55411/version/1/file/Fallpauschalen_Katalog_2018_171124.xlsx> |
|  | Medical devices | Institute for Hospital Remuneration System | <https://www.g-drg.de/G-DRG-System_2019/Fallpauschalen-Katalog/Fallpauschalen-Katalog_2019> |
|  | Health products/ Disposables | Apothekerverband Westfallen-Lippe | <https://expydoc.com/doc/8068217/preisvereinbarung-und-preisliste-zu-blut> |
|  | Personnel | Zi-Praxis-Panel | <https://www.zi-pp.de/pdf/ZiPP_Jahresbericht_2017.pdf> |
|  |  | National Association of Statutory Health Insurance Physicians | <https://www.kbv.de/media/sp/Honorarbericht_Quartal_4_2017.pdf> |
| Composite goods and services | Outpatient visit | National Association of Statutory Health Insurance Physicians | <https://www.dkgev.de/fileadmin/default/Mediapool/2_Themen/2.2_Finanzierung_und_Leistungskataloge/2.2.3._Ambulante_Verguetung/2.2.3.4._Ambulante_Notfallvehandlung_durch_Krankenhaeuser/2015-02-17_Gutachten_zur_ambulanten_Notfallversorgung_im_Krankenhaus_2015.pdf> |
|  | Image diagnosis | National Association of Statutory Health Insurance Physicians | <https://www.kbv.de/media/sp/EBM_Gesamt_-_Stand_3._Quartal_2020.pdf> |
|  | Laboratory tests | National Association of Statutory Health Insurance Physicians | <https://www.kbv.de/media/sp/EBM_Gesamt_-_Stand_3._Quartal_2020.pdf> |
|  | Ambulance services | City administration of Königswinter | <http://www.witten.de/fileadmin/user_upload/Dokumente/sta10/orecht/or3/314.pdf> |
|  | Diagnostic procedures | National Association of Statutory Health Insurance Physicians | https://www.kbv.de/media/sp/EBM_Gesamt_-_Stand_1._Quartal_2019.pdf |
|  | Therapeutic procedures | Institute for Hospital Remuneration System | <https://www.g-drg.de/G-DRG-System_2020/Fallpauschalen-Katalog/Fallpauschalen-Katalog_2020> |
|  |  | National Association of Statutory Health Insurance Physicians | <https://www.kbv.de/media/sp/EBM_Gesamt_-_Stand_2._Quartal_2020.pdf> |
| Complex processes and interventions | Inpatient medical and surgical processes | Institute for Hospital Remuneration System | <https://www.g-drg.de/G-DRG-System_2020/Fallpauschalen-Katalog/Fallpauschalen-Katalog_2020> |
|  | Day case procedures/ Outpatient surgery | Institute for Hospital Remuneration System | <https://www.g-drg.de/G-DRG-System_2020/Fallpauschalen-Katalog/Fallpauschalen-Katalog_2020> |
|  |  | North Rhine Association of Statutory Health Insurance Physicians | <https://www.kvno.de/fileadmin/shared/pdf/online/vertraege/katarakt/katarakt_vdek.pdf> |

| **Italy** | | | |
| --- | --- | --- | --- |
| **Category** | **Subcategory** | **Institution that publishes the cost** | **Link to the website where the cost is published** |
| Primary resources | Medicines | Italian Medicines Agency | https://www.aifa.gov.it/liste-farmaci-a-h |
|  | Medical devices | Italian Ministry of Health | https://www.gazzettaufficiale.it/atto/serie_generale/caricaDettaglioAtto/originario?atto.dataPubblicazioneGazzetta=2007-11-13&atto.codiceRedazionale=07A09617&elenco30giorni=false |
|  |  | National Anti-Corruption Authority | <https://www.anticorruzione.it/portal/rest/jcr/repository/collaboration/Digital%20Assets/anacdocs/Attivita/Pubblicazioni/RapportiStudi/Disp.medici09.07.2013.note_TAR%20.pdf> |
|  |  | Italian provinces’ Ministries of Health | <https://www.asst-mantova.it/documents/338413/5738339/Esito+%28327%29.pdf/7579a98d-eba5-89f6-2bd0-4658ed326526> |
|  |  |  | <http://www.asp.enna.it/portale/attachments/article/3155/determina%20n.%20243%20del%2021.03.2017.pdf> |
|  |  |  | <https://www.aobrotzu.it/documenti/9_204_20190124111717.pdf> |
|  | Health products/ Disposables | Italian provinces’ Ministries of Health | https://www.asl1.liguria.it/download.asp?id=38180 |
|  |  |  | https://www.regione.veneto.it/web/sanita/prescrizione-e-dispensazione-dispositivi-per-diabetici |
|  | Personnel | Italian Doctors Union | <http://www.quotidianosanita.it/allegati/allegato4913967.pdf> |
|  |  | National Collective Agreement | <http://www.medicoeleggi.com/argomenti00/italia2006/acn2005-59.htm> |
|  |  | National Institute of Statistics | <https://www.istat.it/it/archivio/194951> |
| Composite goods and services | Outpatient visit | Italian Ministry of Health | https://www.gazzettaufficiale.it/eli/id/2013/01/28/13A00528/sg |
|  |  | Italian provinces’ Ministries of Health | http://www.mattoni.salute.gov.it/mattoni/documenti/11_Valutazione_costi_dell_emergenza.pdf |
|  |  |  | http://www.regione.piemonte.it/sanita/cms2/component/phocadownload/category/68-nomenclatore-tariffario-regionale-delle-prestazioni-di-assistenza-specialistico-ambulatoriali.html |
|  | Image diagnosis | Italian Ministry of Health | https://www.gazzettaufficiale.it/eli/id/2013/01/28/13A00528/sg |
|  |  | Italian provinces’ Ministries of Health | http://www.regione.piemonte.it/sanita/cms2/component/phocadownload/category/68-nomenclatore-tariffario-regionale-delle-prestazioni-di-assistenza-specialistico-ambulatoriali.html |
|  | Laboratory tests | Italian Ministry of Health | https://www.gazzettaufficiale.it/eli/id/2013/01/28/13A00528/sg |
|  |  | Italian provinces’ Ministries of Health | http://www.regione.piemonte.it/sanita/cms2/component/phocadownload/category/68-nomenclatore-tariffario-regionale-delle-prestazioni-di-assistenza-specialistico-ambulatoriali.html |
|  | Ambulance services | Italian provinces’ Ministries of Health | http://www.mattoni.salute.gov.it/mattoni/documenti/11_Valutazione_costi_dell_emergenza.pdf |
|  | Diagnostic procedures | Italian Ministry of Health | https://www.gazzettaufficiale.it/eli/id/2013/01/28/13A00528/sg |
|  | Therapeutic procedures | Italian Ministry of Health | https://www.gazzettaufficiale.it/eli/id/2013/01/28/13A00528/sg |
|  |  | Italian provinces’ Ministries of Health | http://www.regione.piemonte.it/sanita/cms2/component/phocadownload/category/68-nomenclatore-tariffario-regionale-delle-prestazioni-di-assistenza-specialistico-ambulatoriali.html |
| Complex processes and interventions | Inpatient medical and surgical processes | Italian Ministry of Health | https://www.gazzettaufficiale.it/eli/id/2013/01/28/13A00528/sg |
|  | Day case procedures/ Outpatient surgery |  |  |

| **Poland** | | | |
| --- | --- | --- | --- |
| **Category** | **Subcategory** | **Institution that publishes the cost** | **Link to the website where the cost is published** |
| Primary resources | Medicines | National Health Fund | <https://www.gov.pl/web/zdrowie/obwieszczenie-ministra-zdrowia-z-dnia-30-kwietnia-2019-r-w-sprawie-wykazu-refundowanych-lekow-srodkow-spozywczych-specjalnego-przeznaczenia-zywieniowego-oraz-wyrobow-medycznych-na-1-maja-2019-r> |
|  | Health products/ Disposables | National Health Fund | <https://www.gov.pl/web/zdrowie/obwieszczenie-ministra-zdrowia-z-dnia-30-kwietnia-2019-r-w-sprawie-wykazu-refundowanych-lekow-srodkow-spozywczych-specjalnego-przeznaczenia-zywieniowego-oraz-wyrobow-medycznych-na-1-maja-2019-r> |
|  | Personnel | National Health Fund | http://nfz.gov.pl/zarzadzenia-prezesa/zarzadzenia-prezesa-nfz/zarzadzenie-nr-1202018dsoz,6844.html |
| Composite goods and services | Outpatient visit | National Health Fund | http://www.nfz.gov.pl/zarzadzenia-prezesa/zarzadzenia-prezesa-nfz/zarzadzenie-nr-222018dsoz-tekst-ujednolicony,6924.html |
|  | Hospitalization |  | http://www.nfz.gov.pl/zarzadzenia-prezesa/zarzadzenia-prezesa-nfz/zarzadzenie-nr-382019dsoz,6906.html |
|  | Image diagnosis |  | http://www.nfz.gov.pl/zarzadzenia-prezesa/zarzadzenia-prezesa-nfz/zarzadzenie-nr-222018dsoz-tekst-ujednolicony,6924.html |
|  | Ambulance services |  | http://nfz.gov.pl/zarzadzenia-prezesa/zarzadzenia-prezesa-nfz/zarzadzenie-nr-1202018dsoz,6844.html |
|  | Diagnostic procedures |  | http://www.nfz.gov.pl/zarzadzenia-prezesa/zarzadzenia-prezesa-nfz/zarzadzenie-nr-222018dsoz-tekst-ujednolicony,6924.html |
|  | Therapeutic procedures |  | http://www.nfz.gov.pl/zarzadzenia-prezesa/zarzadzenia-prezesa-nfz/zarzadzenie-nr-222018dsoz-tekst-ujednolicony,6924.html |
|  |  |  | http://www.nfz.gov.pl/zarzadzenia-prezesa/zarzadzenia-prezesa-nfz/zarzadzenie-nr-452019dsoz,6912.html |
| Complex processes and interventions | Inpatient medical and surgical processes | National Health Fund | http://www.nfz.gov.pl/zarzadzenia-prezesa/zarzadzenia-prezesa-nfz/zarzadzenie-nr-222018dsoz-tekst-ujednolicony,6924.html |
|  |  |  | http://www.nfz.gov.pl/zarzadzenia-prezesa/zarzadzenia-prezesa-nfz/zarzadzenie-nr-382019dsoz,6906.html |
|  | Day case procedures/ Outpatient surgery |  | http://www.nfz.gov.pl/zarzadzenia-prezesa/zarzadzenia-prezesa-nfz/zarzadzenie-nr-382019dsoz,6906.html |

| **Portugal** | | | |
| --- | --- | --- | --- |
| **Category** | **Subcategory** | **Institution that publishes the cost** | **Link to the website where the cost is published** |
| Primary resources | Medicines | INFARMED | http://app10.infarmed.pt/genericos/genericos_II/lista_genericos.php?tabela=dispt&fonte=dci&escolha_dci=QXRvcnZhc3RhdGluYQ== |
|  |  |  | http://app10.infarmed.pt/genericos/genericos_II/lista_genericos.php?tabela=dispt&fonte=dci&escolha_dci=UGFyYWNldGFtb2w= |
|  |  | Setúbal Hospital Centre | http://www.base.gov.pt/base2/rest/documentos/737159 |
|  | Medical devices | National Health Service | http://www.base.gov.pt/base2/rest/documentos/554949 |
|  |  |  | http://www.base.gov.pt/base2/rest/documentos/157090 |
|  | Health products/ Disposables | National Health Service | http://www.base.gov.pt/base2/rest/documentos/380625 |
|  | Personnel | Independent Physicians Trade Union | https://www.simedicos.pt/fotos/editor2/ficheiros/tabela_salarial_2019.pdf |
|  |  | Portuguese Nurses Trade Union | https://www.sep.org.pt/files/uploads/2017/06/sep_23062017_TSEnfermagem_2017_35horas_a_partir_1abril.pdf |
| Composite goods and services | Outpatient visit | Central Administration of the Health System | http://www.acss.min-saude.pt/wp-content/uploads/2017/11/Termos-Referencia-Contratualizacao-SNS_2018.pdf |
|  |  | Portuguese Court of Auditors | https://www.tcontas.pt/pt/actos/rel_auditoria/2014/2s/audit-dgtc-rel017-2014-2s.pdf |
|  | Hospitalization | Regulatory Health Authority | https://www.ers.pt/uploads/writer_file/document/1010/ERS_-_Parecer_Limites_Pre_os_SNS__1.Abr.2014__pub.pdf |
|  |  | National Health Service | https://www.sns.gov.pt/wp-content/uploads/2016/05/Avalia%C3%A7%C3%A3o-nacional-da-situa%C3%A7%C3%A3o-das-unidades-de-cuidados-intensivos.pdf |
|  | Image diagnosis | National Health Service | http://www.acss.min-saude.pt/category/acss_pt/tabelas-e-impressos/ |
|  | Laboratory tests | National Health Service | http://www.acss.min-saude.pt/category/acss_pt/tabelas-e-impressos/ |
|  | Ambulance services | Regulatory Health Authority | https://www.ers.pt/uploads/writer_file/document/108/200731583312842202_original_rel.pdf |
|  |  | National Health Service | https://dre.pt/web/guest/pesquisa/-/search/924995/details/normal?q=Despacho+n.%C2%BA%2019965%2F2008 |
|  | Diagnostic procedures | National Health Service | http://www.acss.min-saude.pt/category/acss_pt/tabelas-e-impressos/ |
|  | Therapeutic procedures | National Health Service | https://dre.pt/web/guest/pesquisa/-/search/106955056/details/normal?q=3668-b%2F2017 |
|  |  |  | http://www.acss.min-saude.pt/category/acss_pt/tabelas-e-impressos/ |
| Complex processes and interventions | Inpatient medical and surgical processes | National Health Service | http://www.acss.min-saude.pt/category/acss_pt/tabelas-e-impressos/ |
|  | Day case procedures/ Outpatient surgery |  |  |

| **Slovenia** | | | |
| --- | --- | --- | --- |
| **Category** | **Subcategory** | **Institution that publishes the cost** | **Link to the website where the cost is published** |
| Primary resources | Medicines | Agency for Medicinal Products and Medical Devices of the Republic of Slovenia | https://www.jazmp.si/fileadmin/datoteke/seznami/SFE/Cene/cene_2007hist.html |
|  | Medical devices | Ministry of Public Administration of the Republic of Slovenia | https://www.enarocanje.si/objavaPogodb/PogodbaDetajli.aspx?IDPogodbeZaceten=72833 |
|  | Health products/ Disposables | Health Insurance Institute of Slovenia | http://www.zzzs.si/egradivap/DFDC914987E44E2AC1257353003EC73A |
| Composite goods and services | Outpatient visit | Health Insurance Institute of Slovenia | http://www.zzzs.si/Zzzs/info/egradiva.nsf/o/37D1B2F27B0EC343C12583B7002DAF04?OpenDocument |
|  | Image diagnosis |  | http://www.zzzs.si/Zzzs/info/egradiva.nsf/o/37D1B2F27B0EC343C12583B7002DAF04?OpenDocument |
|  | Laboratory tests | Health Insurance Institute of Slovenia | http://www.zzzs.si/Zzzs/info/egradiva.nsf/o/37D1B2F27B0EC343C12583B7002DAF04?OpenDocument |
|  |  | *Zdravstveni dom (ZD) Trebnje* | https://zd-tr.si/cenik-laboratorijskih-storitev-za-samoplacnike |
|  | Ambulance services | Health Insurance Institute of Slovenia | http://www.zzzs.si/Zzzs/info/egradiva.nsf/o/37D1B2F27B0EC343C12583B7002DAF04?OpenDocument |
|  | Diagnostic procedures | Health Insurance Institute of Slovenia | http://www.zzzs.si/Zzzs/info/egradiva.nsf/o/37D1B2F27B0EC343C12583B7002DAF04?OpenDocument |
|  | Therapeutic procedures |  |  |
| Complex processes and interventions | Inpatient medical and surgical processes | Health Insurance Institute of Slovenia | https://partner.zzzs.si/wps/portal/portali/aizv/zdravstvene_storitve/plan_in_realizacija/podatki_o_planu_in_realizaciji_zdrav_storitve |
|  | Day case procedures/ Outpatient surgery | Health Insurance Institute of Slovenia | https://partner.zzzs.si/wps/portal/portali/aizv/zdravstvene_storitve/plan_in_realizacija/podatki_o_planu_in_realizaciji_zdrav_storitve |
|  |  |  | http://www.zzzs.si/Zzzs/info/egradiva.nsf/o/37D1B2F27B0EC343C12583B7002DAF04?OpenDocument |

| **Spain** | | | |
| --- | --- | --- | --- |
| **Category** | **Subcategory** | **Institution that publishes the cost** | **Link to the website where the cost is published** |
| Primary resources | Medicines | BotPlus | https://botplusweb.portalfarma.com/botplus.aspx |
|  | Health products/ Disposables | BotPlus | https://botplusweb.portalfarma.com/botplus.aspx |
| Composite goods and services | Outpatient visit | Healthcare systems of Autonomous Regions of Spain | - https://www.osakidetza.euskadi.eus/contenidos/informacion/libro_tarifas/es_libro/adjuntos/tarifas_2019.pdf - <https://www.xunta.gal/dog/Publicados/2017/20170714/AnuncioG0164-100717-0001_es.html> - <http://www.madrid.org/wleg_pub/secure/normativas/contenidoNormativa.jsf?opcion=VerHtml&nmnorma=9930&cdestado=P#no-back-button> - <https://www3.gobiernodecanarias.org/sanidad/scs/contenidoGenerico.jsp?idDocument=169e14ff-4f56-11e7-a85b-271b608162d1&idCarpeta=08d3bd15-af33-11dd-a7d2-0594d2361b6c> - <http://www.boa.aragon.es/cgi-bin/EBOA/BRSCGI?CMD=VEROBJ&MLKOB=977342223030> - <https://www.boe.es/boe/dias/2013/07/29/pdfs/BOE-A-2013-8240.pdf> |
|  | Hospitalization | Healthcare systems of Autonomous Regions of Spain | - <https://juntadeandalucia.es/boja/2016/218/BOJA16-218-00003-19739-01_00102029.pdf> - <https://www.xunta.gal/dog/Publicados/2017/20170714/AnuncioG0164-100717-0001_es.html> - <https://www3.gobiernodecanarias.org/sanidad/scs/contenidoGenerico.jsp?idDocument=169e14ff-4f56-11e7-a85b-271b608162d1&idCarpeta=08d3bd15-af33-11dd-a7d2-0594d2361b6c> - <http://www.boa.aragon.es/cgi-bin/EBOA/BRSCGI?CMD=VEROBJ&MLKOB=977342223030> - <https://www.osakidetza.euskadi.eus/contenidos/informacion/libro_tarifas/es_libro/adjuntos/tarifas_2019.pdf> |
|  | Image diagnosis | Healthcare systems of Autonomous Regions of Spain | - <https://www3.gobiernodecanarias.org/sanidad/scs/contenidoGenerico.jsp?idDocument=169e14ff-4f56-11e7-a85b-271b608162d1&idCarpeta=08d3bd15-af33-11dd-a7d2-0594d2361b6c> - <http://www.boa.aragon.es/cgi-bin/EBOA/BRSCGI?CMD=VEROBJ&MLKOB=977342223030> - <https://www.osakidetza.euskadi.eus/contenidos/informacion/libro_tarifas/es_libro/adjuntos/tarifas_2019.pdf> - <https://www.xunta.gal/dog/Publicados/2017/20170714/AnuncioG0164-100717-0001_es.html> - <http://www.madrid.org/wleg_pub/secure/normativas/contenidoNormativa.jsf?opcion=VerHtml&nmnorma=9930&cdestado=P#no-back-button> - https://juntadeandalucia.es/boja/2005/210/d28.pdf |
|  | Laboratory tests | Healthcare systems of Autonomous Regions of Spain | - <https://www.osakidetza.euskadi.eus/contenidos/informacion/libro_tarifas/es_libro/adjuntos/tarifas_2019.pdf> - https://juntadeandalucia.es/boja/2005/210/d28.pdf |
|  | Ambulance services | Healthcare systems of Autonomous Regions of Spain | - <http://www.madrid.org/wleg_pub/secure/normativas/contenidoNormativa.jsf?opcion=VerHtml&nmnorma=9930&cdestado=P#no-back-button> - <https://www.xunta.gal/dog/Publicados/2017/20170714/AnuncioG0164-100717-0001_es.html> - <https://www.osakidetza.euskadi.eus/contenidos/informacion/libro_tarifas/es_libro/adjuntos/tarifas_2019.pdf> - https://juntadeandalucia.es/boja/2005/210/d28.pdf |
|  | Diagnostic procedures | Healthcare systems of Autonomous Regions of Spain | - <https://www.osakidetza.euskadi.eus/contenidos/informacion/libro_tarifas/es_libro/adjuntos/tarifas_2019.pdf> - <https://www.xunta.gal/dog/Publicados/2017/20170714/AnuncioG0164-100717-0001_es.html> - <https://juntadeandalucia.es/boja/2005/210/d28.pdf> - <https://www3.gobiernodecanarias.org/sanidad/scs/contenidoGenerico.jsp?idDocument=169e14ff-4f56-11e7-a85b-271b608162d1&idCarpeta=08d3bd15-af33-11dd-a7d2-0594d2361b6c> - <http://www.madrid.org/wleg_pub/secure/normativas/contenidoNormativa.jsf?opcion=VerHtml&nmnorma=9930&cdestado=P#no-back-button> - http://www.boa.aragon.es/cgi-bin/EBOA/BRSCGI?CMD=VEROBJ&MLKOB=977342223030 |
|  | Therapeutic procedures |  |  |
| Complex processes and interventions | Inpatient medical and surgical processes | National Health Service | <https://www.mscbs.gob.es/estadEstudios/estadisticas/inforRecopilaciones/anaDesarrolloGDR.htm> |
|  |  | Healthcare systems of Autonomous Regions of Spain | - <https://www.osakidetza.euskadi.eus/contenidos/informacion/libro_tarifas/es_libro/adjuntos/tarifas_2019.pdf> - <http://www.madrid.org/wleg_pub/secure/normativas/contenidoNormativa.jsf?opcion=VerHtml&nmnorma=9930&cdestado=P#no-back-button> - <https://juntadeandalucia.es/boja/2005/210/d28.pdf> - http://www.boa.aragon.es/cgi-bin/EBOA/BRSCGI?CMD=VEROBJ&MLKOB=977342223030 |
|  | Day case procedures/ Outpatient surgery | National Health Service | <https://www.mscbs.gob.es/estadEstudios/estadisticas/inforRecopilaciones/anaDesarrolloGDR.htm> |
|  |  | Healthcare systems of Autonomous Regions of Spain | - <http://www.boa.aragon.es/cgi-bin/EBOA/BRSCGI?CMD=VEROBJ&MLKOB=977342223030> - <http://www.madrid.org/wleg_pub/secure/normativas/contenidoNormativa.jsf?opcion=VerHtml&nmnorma=9930&cdestado=P#no-back-button> - <https://juntadeandalucia.es/boja/2005/210/d28.pdf> - <https://www.xunta.gal/dog/Publicados/2017/20170714/AnuncioG0164-100717-0001_es.html> - https://www3.gobiernodecanarias.org/sanidad/scs/contenidoGenerico.jsp?idDocument=169e14ff-4f56-11e7-a85b-271b608162d1&idCarpeta=08d3bd15-af33-11dd-a7d2-0594d2361b6c |

| **Sweden** | | | |
| --- | --- | --- | --- |
| **Category** | **Subcategory** | **Institution that publishes the cost** | **Link to the website where the cost is published** |
| Primary resources | Medicines | Dental and Pharmaceutical Benefits Agency | https://tlv.se/beslut/sok-i-databasen.html |
|  | Health products/ Disposables |  | https://varuforsorjningen.se/artiklar/p-glukos-snabbtest/blodsticka-glukos-ej-dalarna-49686 |
| Composite goods and services | Outpatient visit | Southern Healthcare Region | https://sodrasjukvardsregionen.se/download/regionala-priser-och-ersattningar-for-sodra-sjukvardsregionen-2020/ |
|  |  | South-eastern healthcare region | https://plus.rjl.se/infopage.jsf?nodeId=41089 |
|  | Hospitalization | Southern Healthcare Region | https://sodrasjukvardsregionen.se/download/regionala-priser-och-ersattningar-for-sodra-sjukvardsregionen-2020/ |
|  | Image diagnosis | Southern Healthcare Region | https://sodrasjukvardsregionen.se/download/regionala-priser-och-ersattningar-for-sodra-sjukvardsregionen-2020/ |
|  |  | South-eastern healthcare region | https://plus.rjl.se/infopage.jsf?nodeId=41089 |
|  | Laboratory tests | Southern Healthcare Region | https://vardgivare.skane.se/patientadministration/avgifter-och-prislistor/prislistor-laboratoriemedicin/ |
|  | Ambulance services | Southern Healthcare Region | https://sodrasjukvardsregionen.se/download/regionala-priser-och-ersattningar-for-sodra-sjukvardsregionen-2020/ |
|  |  | South-eastern healthcare region | https://plus.rjl.se/infopage.jsf?nodeId=41089 |
|  | Diagnostic procedures | Southern Healthcare Region | https://sodrasjukvardsregionen.se/download/regionala-priser-och-ersattningar-for-sodra-sjukvardsregionen-2020/ |
|  |  | South-eastern healthcare region | https://plus.rjl.se/infopage.jsf?nodeId=41089 |
|  | Therapeutic procedures | Southern Healthcare Region | https://sodrasjukvardsregionen.se/download/regionala-priser-och-ersattningar-for-sodra-sjukvardsregionen-2020/ |
| Complex processes and interventions | Inpatient medical and surgical processes | Southern Healthcare Region | https://sodrasjukvardsregionen.se/download/regionala-priser-och-ersattningar-for-sodra-sjukvardsregionen-2020/ |
|  |  | South-eastern healthcare region | https://plus.rjl.se/infopage.jsf?nodeId=41089 |
|  | Day case procedures/ Outpatient surgery | Southern Healthcare Region | https://sodrasjukvardsregionen.se/download/regionala-priser-och-ersattningar-for-sodra-sjukvardsregionen-2020/ |
|  |  | South-eastern healthcare region | https://plus.rjl.se/infopage.jsf?nodeId=41089 |
